# Efavirenz Repurposing Challenges: A Novel Nanomicelle-Based Antiviral Therapy Against Mosquito-Borne Flaviviruses

**DOI:** 10.3390/pharmaceutics17020241

**Published:** 2025-02-12

**Authors:** Sofía Maldonado, Pedro Fuentes, Ezequiel Bernabeu, Facundo Bertera, Javier Opezzo, Eduardo Lagomarsino, Hyun J. Lee, Fleming Martínez Rodríguez, Marcelo R. Choi, María Jimena Salgueiro, Elsa B. Damonte, Christian Höcht, Marcela A. Moretton, Claudia S. Sepúlveda, Diego A. Chiappetta

**Affiliations:** 1 Departamento de Tecnología Farmacéutica, Facultad de Farmacia y Bioquímica, Universidad de Buenos Aires, Buenos Aires CP1113, Argentina; sofiamaldonado@qb.fcen.uba.ar (S.M.); pfuentes@docente.ffyb.uba.ar (P.F.); ebernabeu@ffyb.uba.ar (E.B.); diegochiappetta@yahoo.com.ar (D.A.C.); 2 Instituto de Tecnología Farmacéutica y Biofarmacia (InTecFyB), Universidad de Buenos Aires, Buenos Aires CP1113, Argentina; fbertera@ffyb.uba.ar (F.B.); jopezzo@ffyb.ub.ar (J.O.); elagomarsino@ffyb.uba.ar (E.L.); jsalgueiro@ffyb.uba.ar (M.J.S.); chocht@ffyb.uba.ar (C.H.); 3 Consejo Nacional de Investigaciones Científicas y Técnicas (CONICET), Buenos Aires CP1113, Argentina; 4 Departamento de Farmacología, Facultad de Farmacia y Bioquímica, Universidad de Buenos Aires, Buenos Aires CP1113, Argentina; 5 Departamento de Farmacia Clínica, Facultad de Farmacia y Bioquímica, Universidad de Buenos Aires, Buenos Aires CP1113, Argentina; 6 Departamento de Ciencias Biológicas, Facultad de Farmacia y Bioquímica, Universidad de Buenos Aires, Buenos Aires CP1113, Argentina; glee@ffyb.uba.ar (H.J.L.); mchoi@ffyb.uba.ar (M.R.C.); 7 Grupo de Investigaciones Farmacéutico-Fisicoquímicas, Departamento de Farmacia, Facultad de Ciencias, Universidad Nacional de Colombia, Sede Bogotá, Bogotá 111321, Colombia; fmartinezr@unal.edu.co; 8 Instituto Alberto C. Taquini de Investigaciones en Medicina Traslacional (IATIMET), CONICET, Universidad de Buenos Aires, Buenos Aires CP1113, Argentina; 9 Departamento de Fisicomatemática, Facultad de Farmacia y Bioquímica, Universidad de Buenos Aires, Buenos Aires CP1113, Argentina; 10 Instituto de Química Biológica de la Facultad de Ciencias Exactas y Naturales (IQUIBICEN), CONICET, Universidad de Buenos Aires, Buenos Aires CP1113, Argentina; edamonte@qb.fcen.uba.ar (E.B.D.); claudia@qb.fcen.uba.ar (C.S.S.)

**Keywords:** efavirenz, drug repurposing, polymeric micelles, dengue, Zika, yellow fever

## Abstract

**Background/Objective:** World Health Organization latest statistics state that 17% of infectious diseases are transmitted by vectors, causing more than 700,000 deaths each year. Particularly, dengue (DENV), Zika (ZIKV) and yellow fever (YFV) viral infections have generated international awareness due to their epidemic proportion and risks of international spread. In this framework, the repositioning strategy of Efavirenz (EFV) represents a key clinical feature to improve different antiviral therapies. Therefore, the development of Soluplus^®^-based nanomicelles (NMs) loaded with EFV (10 mg/mL) for optimized oral pharmacotherapy against ZIKV, DENV and YFV infections was investigated. **Methods:** EFV-NMs were obtained by an acetone diffusion technique. Micellar size and in vitro micellar interaction with mucin were assessed by dynamic light scattering. In vitro cytocompatibility was investigated in A549 and Vero cells and micellar in vitro antiviral activity against ZIKV, DENV and YFV was evaluated. In vivo oral bioavailability and histological studies were assessed in Wistar rats. **Results:** EFV encapsulation within Soluplus^®^ NMs increased the drug’s apparent aqueous solubility up to 4803-fold with a unimodal micellar size distribution and a micellar size of ~90 nm at 25 and 37 °C. Micellar in vitro interaction with mucin was also assessed in a pH range of 1.2–7.5 and its storage micellar physicochemical stability at 4 °C was confirmed over 2 years. In vitro cytocompatibility assays in A549 and Vero cells confirmed that EFV micellar dispersions resulted in safe nanoformulations. Interestingly, EFV-loaded NMs exhibited significantly higher in vitro antiviral activity compared with EFV solution for all the tested flaviviruses. In addition, the selectivity index (SI) values reveal that EFV-loaded NMs exhibited considerably more biological efficacy compared to EFV solution in A549 and Vero cell lines and for each viral infection (SI > 10). Further, the drug pharmacokinetics parameters were enhanced after the oral administration of EFV-loaded NMs, being biocompatible by not causing damage in the gastrointestinal segments. **Conclusions:** Overall, our EFV nanoformulation highlighted its potential as a novel drug delivery platform for optimized ZIKV, DENV and YFV antiviral therapy.

## 1. Introduction

According to the World Health Organization (WHO), 17% of infectious diseases are transmitted by vectors, causing more than 700,000 deaths each year [1]. These diseases are caused by parasites, bacteria or viruses. International concern has been generated with the emergence and re-emergence of viral diseases such as dengue, Zika, chikungunya and yellow fever over the past 5 decades [2]. Particularly, dengue fever is caused by dengue virus (DENV), and it is currently the most common infection transmitted by mosquitoes of the *Aedes* genus. About half of the world’s population is at risk of contracting dengue, and actualized estimations suggest that there are between 100 and 400 million infections each year [3]. Approximately 1 in 20 patients with dengue progress to develop a severe, life-threatening disease called severe dengue [4]. This disease has a mortality rate of less than 1% if managed properly, but can reach 4% in severe untreated cases [5]. In 2016, the WHO declared Zika virus (ZIKV) infections and their association with congenital malformations and other neurological disorders an international public health emergency, due to their spread to almost the entire American continent [6]. Between 2015 and 2016, the mortalities of newborns in Brazil with congenital infection by ZIKV and microcephaly were 4–6% and 8.3%, respectively [7]. Since 2019, autochthonous mosquito-borne transmission of ZIKV has been confirmed in 87 countries (tropical and subtropical territories), representing 4 of the 6 WHO Regions [8]. Yellow fever is another infectious disease with epidemic capacity in Africa and South America, with lethality rates in severe cases of up to 60%, despite having an efficacious vaccine [9]. Approximately 15% of people infected with the yellow fever virus (YFV) develop severe forms of the disease, including high fever, jaundice and liver and kidney failure. Among these severe forms, mortality varies between 20 and 50% [10].

Overall, these statistics highlight the fact that these diseases have taken on an epidemic proportion in various parts of the world over the past few years with a risk of international spread, afflicting all populations, claiming thousand lives, and overwhelming health services and infrastructure [1]. This last situation is mainly associated with a combination of different factors such as urbanization, international travel, climate change and variation in mosquito abundance and distribution, facilitating the geographical spread of disease vectors to new geographical areas in the current century [2].

Interestingly, these infections have some things in common; for example, they are caused by a flavivirus, they are transmitted by the same vector (mosquito of the genus *Aedes*) and, so far, they do not present specific antiviral drugs for their treatment.

In the last 50 years, great efforts have been made to discover and design antiviral drugs, some of which have obtained approval for use in infectious diseases. However, to date, no drug has been approved by a regulatory authority for diseases caused by a mosquito-borne flavivirus [11].

The development of new drugs is an expensive process that requires several years of research and development to obtain a final product ready for approval from a regulatory agency [12]. An interesting alternative to overcoming the disadvantages of this traditional strategy is the so-called repurposing (repositioning) of drugs [13], which can shorten the times and decrease the costs for the approval of a drug with a new therapeutic indication [12].

A drug that has received a lot of attention in recent years due to the possibility of being used to treat other diseases is Efavirenz (EFV) [14]. This drug was approved by the FDA in 1998 as a component in the first-line therapeutic cocktail of HIV treatment, and since a few years ago, several clinical trials have been carried out to evaluate its effectiveness in different types of cancer and neurological diseases, such as Alzheimer’s [14]. Furthermore, the potential use of EFV to inhibit the ZIKV in vitro has been evaluated [15].

EFV is a highly lipophilic molecule with an intrinsic aqueous solubility of 4 µg/mL. It undergoes hepatic metabolism, resulting in poor oral bioavailability (between 40 and 45%) [16]. In this framework, to improve the water-solubility and the oral bioavailability of EFV several nanotechnological approaches have been made [17]. One of the most successful has been its encapsulation within polymeric micelles, which has significantly improved the drug’s aqueous solubility and oral bioavailability of EFV [18].

Recently, our group has demonstrated the potential of an oral nanoformulation based on EFV and curcumin co-encapsulation within Soluplus^®^ NMs for anti-HIV therapy [19]. This biopolymer has demonstrated excellent colloidal stability and biocompatibility. Taking into account the potential of EFV to be used in the treatment of different pathologies, we aimed to explore the in vitro antiviral activity of a micellar nanoformulation based on EFV versus ZIKV, DENV and YFV. With this in mind, EFV-loaded Soluplus^®^ NMs were prepared and characterized in detail. Micellar in vitro interaction with mucin was also assessed and the storage micellar physicochemical stability at 4 °C was evaluated over 2 years. Subsequently, the in vitro cell compatibility and in vitro antiviral activity of EFV-loaded NMs in two mammalian cell lines (A549 and Vero) was examined. Furthermore, the oral bioavailability and safety of the EFV nanoformulation was evaluated in Wistar rats.

## 2. Materials and Methods

### 2.1. Materials

EFV was from LKM SA laboratories (Buenos Aires, Argentina). Polyvinyl caprolactam (PVC)–polyvinylacetate (PVA)–polyethylene glycol (PEG 6000) (Soluplus^®^, MW 120.000 g/mol) was from BASF (Ludwigshafen, Germany). All solvents were of analytical or high-performance liquid chromatography (HPLC) grade and were used following the manufacturer’s instructions.

### 2.2. Preparation of Free and Drug-Loaded NMs

Free-loaded Soluplus^®^ NMs (1–10% *w*/*v*) were prepared by aqueous dispersion of the appropriate amount of copolymer in distilled water under magnetic stirring (500 rpm) at 25 °C for 5 h. Afterwards, samples were equilibrated for 24 h at room temperature before use. Then, an acetone displacement technique [20] was employed to prepare EFV-loaded NMs. Briefly, an excess of EFV was dissolved in acetone (5 mL) and added dropwise with a syringe to an aqueous Soluplus^®^ dispersion (1–10% *w*/*v*, 10 mL) under magnetic stirring (500 rpm) at room temperature overnight to ensure acetone evaporation. Then, the samples were filtered (0.45 μm acetate cellulose filters, Microclar, Buenos Aires, Argentina) to remove insoluble material and stored in closed amber glass vials until use.

High-performance liquid chromatography (RP-HPLC) was employed in order to quantify the concentration of the EFV. In this case, the analytical method included a C18 column (4.6 mm × 250 mm, 5 μm, Fluophase PFP, Thermo, Waltham, MA, USA) with a mobile phase composed of distilled water/acetonitrile/trimethylamine (60:40:0.2 *v*/*v*/*v*; pH 3) and a flow rate of 1.4 mL/min. One hundred microliters of all samples were injected, and the detection wavelength was 248 nm [18].

Solubility factors (F_s_) were then calculated according to Equation (1).

F_s_ = S_a_/S_w_ (1)

where S_a_ represents the apparent solubility of the EFV in the corresponding NM dispersion and S_w_ represents the EFV’s intrinsic solubility in distilled water at 25 °C.

Moreover, data from the solubility studies were used to calculate the following parameters: (i) molar solubilization capacity (X) (moles of drug that can be solubilized per mol of copolymer forming micelles), (ii) micelle/water partition coefficient (P, the ratio between the drug concentration in the micelle and the drug concentration in water), (iii) molar micelle/water partition coefficient (PM) and (iv) the standard free Gibbs energy of solubilization (ΔGs), estimated from the molar micelle/water partition coefficient (PM) using Equations (2)–(5), respectively [21].

X = (S_tot_ − S_w_)/(C_copol_ − CMC) (2)

P = S_tot_ − S_w_/S_w_ (3)

PM = X · (1 − CMC)/S_w_ (4)

ΔGs = −RT · ln(PM) (5)

where S_tot_ represents EFV solubility in the micellar dispersion, S_w_ is the EFV’s intrinsic solubility in distilled water at 25 °C, CMC is the critical micellar concentration at 25 °C and C_copol_ is the copolymer concentration in each micellar dispersion. R is the universal constant of gases (R = 8.31433 J/mol °K) and T was set at 298.15 °K.

Finally, the drug loading (DL) and encapsulation efficiency (EE) of EFV in the NMs were calculated according to the following equations:

DL (%) = (weight of EFV in the NMs)/(total weight of NMs + EFV) × 100(6)

EE (%) = (weight of EFV in the NMs)/(initial weight of EFV used) × 100(7)

Assays were performed in triplicate and results are expressed as means ± S.D.

### 2.3. Micellar Size, Size Distribution and Morphology of NMs

Micellar size and size distribution of free- and EFV-loaded (10 mg/mL) Soluplus^®^ (7% *w*/*v*) micelles were measured by dynamic light scattering (DLS, scattering angle of θ = 173° to the incident beam, Zetasizer Nano-ZSP, ZEN5600, Malvern Instruments, Malvern, UK) at 25 °C and 37 °C. Samples were equilibrated for 3 min at the corresponding temperature before the measurements. Finally, the results of the Z-average (Z-ave) and polydispersity index (PDI) values were expressed as the average of five measurements ± S.D.

Morphological analysis of EFV (10 mg/mL)-NMs (7% *w*/*v*) was performed, employing transmission electron microscopy (TEM) with a Philips CM-12 TEM apparatus (FEI Company, Eindhoven, The Netherlands). Five microliters of drug-loaded NMs were negatively stained with a phosphotungstic acid solution (5 μL, 1% *w*/*v*) on a clean grid covered with Fomvar film.

### 2.4. In Vitro Micellar Interaction with Mucin

Micellar samples were diluted in simulated gastrointestinal fluids (SGIFs; pH 1.2, 6.8, 7.5 and 6.0) (Table S1) [19] in order to obtain a final Soluplus^®^ concentration of 0.6% *w*/*v*. Then, 1 mL of each diluted micellar system was incubated with 1 mL of mucin dispersion (0.05% *w*/*v*) (from bovine submaxillary gland; Sigma, St. Louis, MO, USA) at room temperature under gentle stirring [22]. Then, an aliquot of each sample was collected to measure micellar size by DLS. The measurements were performed in triplicate at 37 °C. Micellar and mucin dispersions properly diluted in SGIFs were used as controls.

### 2.5. Batch-to-Batch Variability and Storage Stability Studies

Firstly, we assayed the batch-to-batch size variability of our nanoformulation. In this way, different batches of EFV-NMs (7% *w*/*v* Soluplus^®^ and 10 mg/mL EFV) were prepared on different days, and then their size and size distribution were determined at 25 °C.

In a second step, we aimed to assess the long-term physical and chemical stability of the colloidal system. Therefore, EFV-NMs were transferred into sealed glass vials and stored at 4 °C (refrigerator temperature) over 2 years. At different time points (0, 1, 6, 12 and 24 months), samples were monitored for changes in particle size and drug content by means of DLS and RP-HPLC, respectively, and as previously described. Results are expressed as means ± standard deviation (S.D.), *n* = 3.

### 2.6. In Vitro EFV Release

The EFV cumulative in vitro release over time from the polymeric matrixes was evaluated by using a dialysis method in four SGIFs (Table S1). Results were compared with those observed for EFV (10 mg/mL)suspension. For this purpose, EFV (10 mg/mL)-NMs (7% *w*/*v*) were diluted (1/10) with distilled water and incorporated inside the dialysis membranes (Spectra/Por^®^3 Dialysis Membrane; molecular weight cut off = 3500; nominal flat width 18 mm; Waltham, MA, USA). The external release medium (10 mL) was added to Falcon^®^ conical tubes. Then, the dialysis membranes were immersed inside the tubes. Each SGIF employed as external medium contained ethanol (30% *v*/*v*) and Tween 80 (0.5% *v*/*v*) to assess sink conditions [19].

Afterwards, each Falcon^®^ conical tube was placed in a sample rotator (40 rpm; Mini Labroller LabNet rotator, St. Louis, MO, USA) and incubated at 37 ± 0.5 °C for 6 h according to the following protocol to mimic the passage of the nanoformulations through the gastrointestinal tract: (i) 2 h in SGF (pH 1.2), (ii) 2 h in SIF (pH 6.8), (iii) 30 min in SIF (pH 7.5) and (iv) 1.5 h in SCF (pH 6.0).

At every time point (1, 2, 3, 4, 4.5 and 6 h), the complete external release medium was replaced by an equal volume of fresh medium pre-heated to 37 °C. The quantification of the EFV concentration was performed by RP-HPLC, as previously described in Section 2.2. Results are expressed as means ± standard deviation (S.D.), *n* = 3.

### 2.7. Cell Culture and Viruses

A549 (human lung adenocarcinoma, ATCC CCL-185) and Vero (African green monkey kidney, ATCC CCL-81) cells were grown in Eagle’s minimum essential medium (MEM, Gibco™ Thermo Fisher Scientific, Waltham, MA, USA) supplemented with 5% heat-inactivated newborn bovine serum (NBS, Gibco™ Thermo Fisher Scientific, Carlsbad, CA, USA) and 1.5 g/L of sodium bicarbonate at 37 °C in a 5% CO_2_ atmosphere. For the maintenance medium (MM), the serum concentration was reduced to 1.5%.

The experiments were performed using a DENV type-2 (DENV-2) strain NGC, a ZIKV INEVH-116141 strain provided by the National Institute of Human Viral Diseases “Dr. Julio I. Maiztegui” (Pergamino, Argentina) through a transfer agreement and the YFV vaccine strain YF-17D kindly provided by Dr. Konigheim of the Institute of Virology of the Faculty of Medical Sciences, National University of Córdoba (Córdoba, Argentina). Virus stocks were propagated in Vero cells and titrated by plaque-forming unit (PFU assay) quantification assays in the same cell line [23].

### 2.8. In Vitro Cytotoxicity Test

Confluent monolayers of A549 or Vero cells grown in 96-well plates were incubated with different concentrations of EFV, drug-free NMs and EFV-NMs for 48 h. Thereafter, cell viability was determined by the 3-(4,5-dimethylthiazol-2-yl)-2,5-diphenyl tetrazolium bromide (MTT, Sigma-Aldrich, St. Louis, MO, USA) method at a final concentration 0.5 mg/mL. After 2 h of incubation at 37 °C, the supernatants were removed, ethanol was added to each well to solubilize the formazan crystals and absorbance was measured in a multi-mode microplate reader (FLUOstar^®^ Omega, Labtech, Eschelbronn, Germany) at 595 nm. The percentage of cellular viability was determined and the cytotoxic concentration 50 (CC_50_) was calculated by linear regression using GraphPad Prism (v8.0.1) software as the compound concentration required to reduce the MTT signal by 50% compared to cell controls. All determinations were performed in triplicate.

### 2.9. Antiviral Assay

In vitro antiviral activity was determined by a virus yield inhibition assay infecting A549 or Vero cells grown in 24-well plates with ZIKV, YFV or DENV-2 at a multiplicity of infection (m.o.i.) of 0.1 PFU/cell. After 1 h adsorption at 37 °C, inocula were discarded and cells were incubated with MM and different concentrations of EFV solution (EFV), EFV-NMs and drug-free NMs (NMs). At 48 h post-infection (p.i.), supernatants were collected, cooled down and extracellular virus titrated by PFU. The effective concentration 50 (EC_50_) was calculated as the compound concentration required to reduce virus yield by 50% in the treated cultures compared with the viral controls (VCs). All determinations were performed in triplicate. EFV and NMs were used as controls.

Statistical analysis was performed using GraphPad Prism (v8.0.1) software. Comparison of means was tested by one-way or two-way analysis of variance (ANOVA) with Dunnett’s post hoc test or utilizing the t-Student test. Statistical significance was defined as *p* < 0.05 (95% confidence interval).

### 2.10. Oral Bioavailability Study

Animal experiments adhered to the ARRIVE guidelines and were in line with the published Guide for the Care and Use of Laboratory Animals (NIH, 8° Ed., 2011) and local regulations. All animal procedures were approved by the Comité Institucional para el Cuidado y Uso de Animales de Laboratorio of the School of Pharmacy and Biochemistry, University of Buenos Aires with the following ethical approval number: CICUAL-FFyB, REDEC-2021-2792-E-UBA-DCT.

EFV oral pharmacokinetics was assessed in male Wistar rats (220–250 g) fasted overnight (12 h). Briefly, EFV (10 mg/mL)-loaded 7% Soluplus^®^ NMs and EFV (10 mg/mL) suspension were administered by gavage (EFV dose: 40 mg/kg). The extemporaneous drug suspension was prepared, employing a 1% dispersion of carboxymethylcellulose in distilled water.

At every time point (0.5, 1, 1.5, 2, 3, 4, 5, 6 and 8 h), 70 µL of blood samples were obtained from the tail vein after the oral administration of the formulations. It is important to highlight that the total blood volume extracted from the rats did not exceed the maximum recommended amount of 3.5 mL [24]. Thereafter, we assumed that the EFV pharmacokinetic parameters were not affected by the blood volume decrement.

Aliquots were centrifuged (10,000 rpm, 10 min, 4 °C) and plasma samples (10 µL) were prepared by simple deproteinization with 20 µL of acetonitrile for further EFV quantification by RP-HPLC. The analytical method consisted of a C18 column (Phenomenex Luna; 5 mm, 150 mm × 4.60 mm, Torrance, CA, USA) with a mobile phase of distilled water/acetonitrile/triethylamine (50:50:0.2; pH 3). The detection wavelength was 248 nm (UVIS 204, Linear Instruments, Reno, NV, USA) with a flow rate of 1.0 mL/min. The calibration curve was validated between 20 and 5000 ng/mL.

Finally, for the evaluation of in vivo data, a non-compartmental analysis of EFV plasma concentrations was performed using the TOPFIT program (version 2.0, Dr Karl Thomae Gmbh, Schering AG, Gödecke AG, Germany). Thereafter, the following pharmacokinetic parameters were estimated: area under the curve (AUC_0–8_), maximum plasma concentration (C_max_) and time to maximum plasma concentration (t_max_). The pharmacokinetic parameters of EFV were log-transformed for statistical analysis, in order to reduce the heterogeneity of the variance, and further compared by one-way ANOVA and the Bonferroni test as a post hoc test. Statistical tests were performed using GraphPad Prism version 8 for Windows (GraphPad Software, San Diego, CA, USA). Statistical significance was defined as *p* < 0.05.

Finally, the relative oral bioavailability (F_r_) was calculated by taking the ratio between the AUC_0–8_ of the nanomicellar systems and the EFV extemporaneous suspension (reference),

F_r_ (%) = AUC_T_/AUC_R_ × 100 (8)

where AUC_T_ and AUC_R_ are the AUC_0–8_ of the tested (T) sample and the reference (R), respectively.

### 2.11. Histological Study

Histological studies were performed to evaluate morphological changes or damage caused by EFV-NMs on the mucosa of stomach, small intestine and large intestine. For this, male Wistar rats (220–250 g) were randomized into two groups. The first group received saline solution and the second received EFV-NMs at an equivalent EFV dose of 40 mg/kg for seven continuous days. Finally, 24 h after the last administration, all rats were sacrificed. The extracted tissue samples were fixed in 10% formaldehyde, embedded in paraffin, sectioned at 6 µm, and stained with hematoxylin and eosin (H&E) according to standard methods. The histological preparations were analyzed with an optical microscope coupled to a digital camera (Nikon Eclipse E200, Tokyo, Japan). Histological evaluation with H&E continues to be the gold standard technique for the study of tissues [25].

## 3. Results and Discussion

In the past few decades, drug repurposing has emerged as an interesting strategy to overcome some of the disadvantages inherent to the drug discovery, research and development process. This approach can be utilized to re-evaluate drugs already on the market, withdrawn medications, and potential drug candidates that were halted during clinical development due to efficacy or commercial concerns, but not due to safety issues [26].

The lack of drugs approved by health regulatory authorities for diseases caused by flaviviruses requires the search of different strategies. For this reason, and taking into account the potential of EFV as a candidate for drug repositioning [14], and its capacity to in vitro inhibit both Zika and West Nile viruses [15], the evaluation of the activity of this molecule against different flaviviruses such as DENV and YFV results in an interesting approach.

### 3.1. EFV Encapsulation

EFV is already categorized as a class II drug (biopharmaceutical classification system, BCS), taking into account its low aqueous solubility and its capacity to permeate through biological membranes [27]. These (bio)limitations clearly affect its gastrointestinal (GI) absorption, mainly associated with its poor solubility in GI fluids and its oral bioavailability [19]. In this sense, the encapsulation of EFV within NMs should offer the following possibilities: (i) minimizing EFV (bio)limitations, (ii) decreasing EFV cytotoxicity and (iii) improving EFV in vitro antiviral activity against DENV, ZIKV and YFV.

**Scheme 1:**
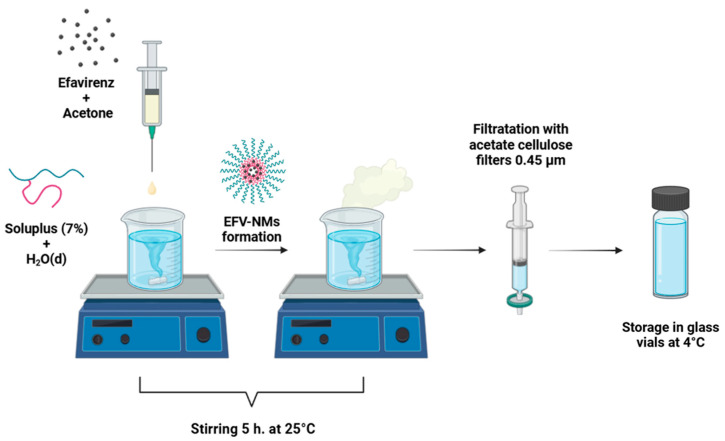
The preparation of EFV-NMs employing an acetone displacement technique. The scheme was made in BioRender (https://biorender.com/, date accessed: 3 January 2025).

**Figure 1:**
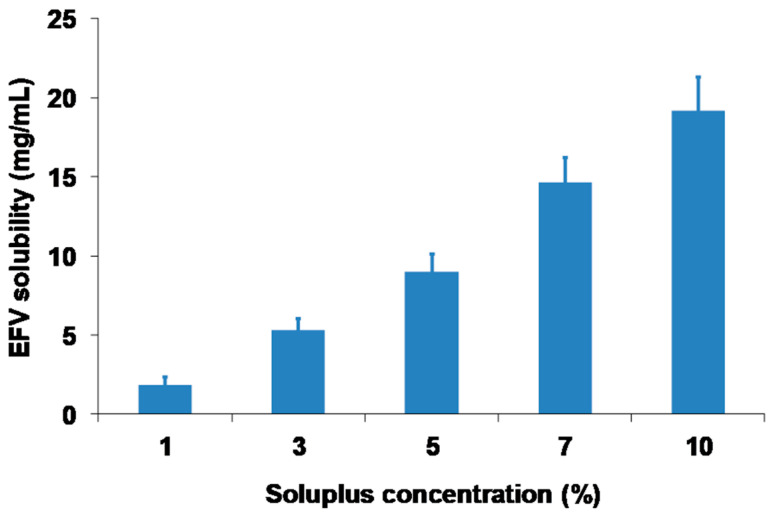
EFV apparent aqueous solubility (S_a_) versus Soluplus^®^ concentration (% *w*/*v*) in distilled water at 25 °C. Results are expressed as mean ± standard deviation (S.D.) (*n* = 3).

In the first place, the acetone displacement technique represents an excellent method to obtain EFV-loaded NMs (Scheme 1). As was expected, the hydrophobic core of the Soluplus^®^ micelles was able to host EFV, denoting a clear increment of the drug’s apparent aqueous solubility. For example, EFV solubility increased from 1.86 (F_s_ = 464) to 19.21 mg/mL (F_s_ = 4803) for 1 and 10% *w*/*v* of Soluplus^®^ colloidal dispersions, respectively (Table 1, Figure 1). In the case of Soluplus^®^, its hydrophobic block is composed of polyvinyl caprolactam and polyvinyl acetate, which serve as an ideal microenvironment for the incorporation of EFV [19]. Also, an increase in the concentration of Soluplus^®^ generates a greater number of micelles that can solubilize a greater amount of the drug [28].

This efficient and spontaneous incorporation of EFV into the nanomicellar core was reflected by the high *p* values and the highly negative ΔGs values (Table 1). Similar increases in EFV solubility were obtained using poloxamer and poloxamine polymeric micelles [16]. Furthermore, with the objective of optimizing our nanoformulation, the estimation of the X and PM parameters of the EFV-NMs was carried out (Table 1). In this context, 7% *w*/*v* of copolymer reached the highest value of the molar micelle/water partition coefficient (6181) and molar solubilization capacity (78.3) (Table 1). These parameters are very important, since they represent the concentration of Soluplus^®^ at which the greatest number of moles of drug per mole of copolymer can be solubilized. Similar behavior was observed for ibuprofen and miconazole Soluplus^®^ micelles [29].

Taking the solubility results into account, we selected the formulation with the best (i) molar solubilization capacity and (ii) molar partition coefficient (EFV-loaded (10 mg/mL) NMs (7% *w*/*v*)) for further studies. This EFV-loaded nanocarrier presents a DL value of 12.4% *w*/*v* and an EE value > 99% (Table 2).

### 3.2. Micellar Size Distribution and Morphology

**Figure 2:**
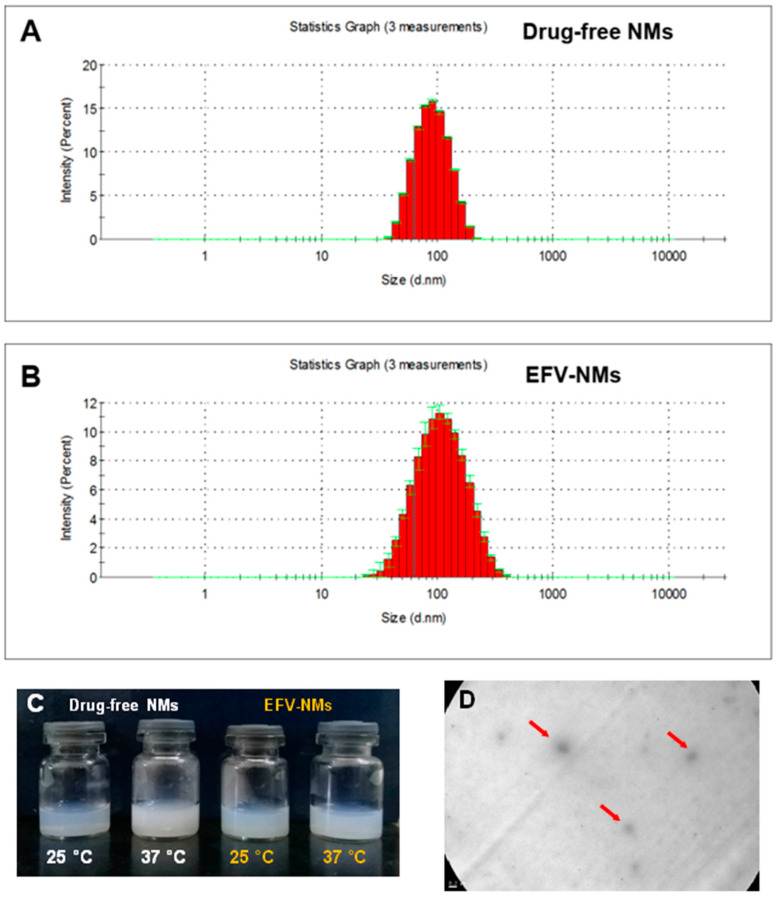
(**A**) Micellar size and size distribution of drug-free NMs and (**B**) EFV-NMs. (**C**) Macroscopic aspect of micellar dispersions, in absence and presence of EFV, at 25 and 37 °C. (**D**) TEM micrographs of EFV-NMs. Red arrows point out NMs (scale bar: 200 nm).

Particle size is one of the most important parameters in nanocarriers, and it has a great impact on the in vivo fate of these systems [30]. With this in mind, the size and size distribution of Soluplus^®^ NMs in absence and presence of EFV at 25 and 37 °C were determined by DLS. At 25 °C, the drug-free NMs exhibited unimodal size distribution with a Z-ave value of 83.4 nm and a PDI value of 0.117. EFV encapsulation presented a slight increment in the NMs size (~1.14-fold), maintaining a unimodal size distribution (Table 2, Figure 2A,B). Similar results were observed for drug-free and acyclovir-loaded Soluplus^®^ micelles [31]. Generally, micellar size is subject to change according to the loading of hydrophobic drugs, thus modifying the aggregation behavior of the micelles [32] and resulting, in some cases, in higher sizes [21,33].

At 37 °C, there was a considerable increase in the Z-ave value (>2 µm) for the drug-free NMs with a PDI value of 1 (Table 2). This behavior is due to the increase in temperature favoring the aggregation of Soluplus^®^ NMs forming a tri-dimensional network structure [34], a phenomenon that can be seen with the naked eye (Figure 2C). PDI values above 0.3 are usually indicative of this phenomenon [28].

On the contrary, a completely different aggregation behavior was observed after EFV encapsulation. A macroscopic aspect revealed the absence of aggregates and/or precipitates where a translucent dispersion was observed (Figure 2C). These results were confirmed by DLS. In this case, the drug-loaded NMs presented a Z-ave value of 85.4 nm with a PDI value of ~0.2 (Table 2). It is clear that the encapsulation of EFV (10 mg/mL) avoids the aggregation of the NMs, preventing the increase in Z-ave and PDI values. Therefore, the average body temperature should not influence the behavior of aggregation of EFV-loaded NMs.

Finally, the morphology of EFV-loaded NMs was visualized by TEM, as shown in Figure 2D. As was expected, the results showed that the nanoformulation exhibited a spherical morphology. These results are in good accordance with the morphology observed for other Soluplus^®^-based NMs [19,33].

### 3.3. In Vitro Micellar Interaction with Mucin

**Figure 3:**
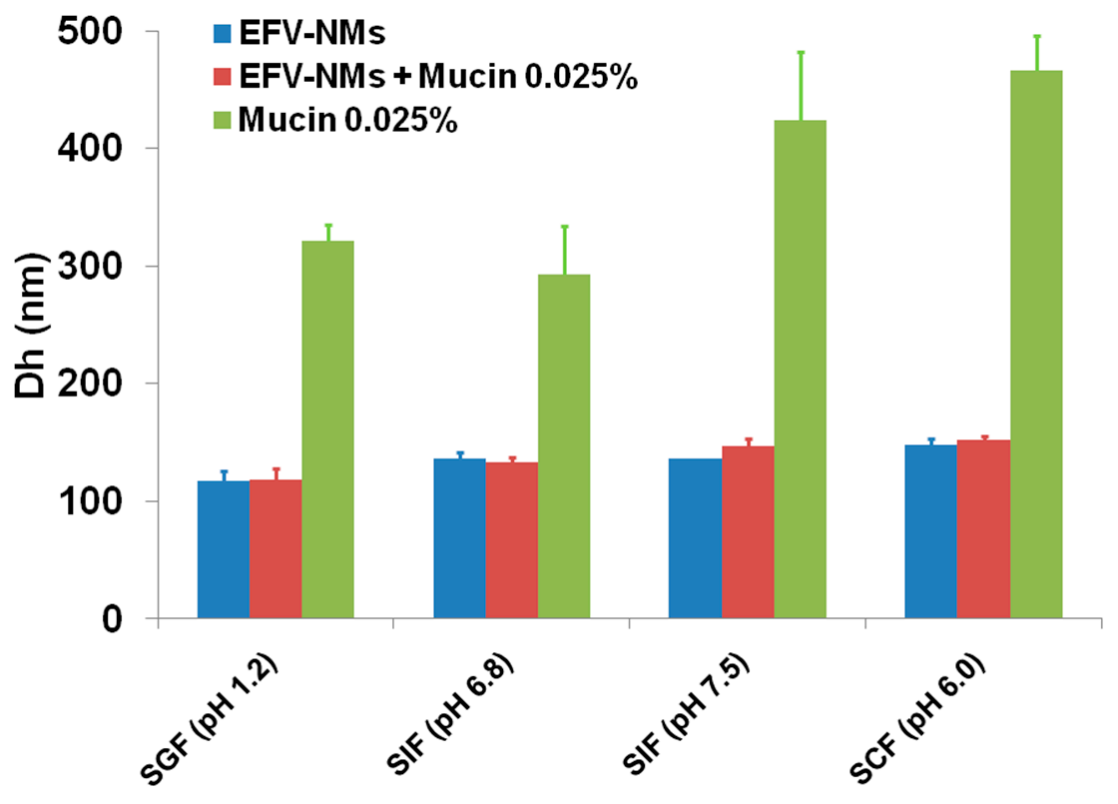
The particle size of the EFV-NMs in the absence and the presence of 0.025% *w*/*v* mucin in different simulated gastrointestinal fluids (pH 1.2, 6.8, 7.5 and 6.0).

The main organ in the gastrointestinal tract responsible for oral drug absorption is the small intestine [35]. This organ has a physical barrier formed by mucus, which protects the intestinal epithelium from harmful substances and pathogens [36], also restricting the passage of nutrients and drugs [37]. Different strategies have been employed to penetrate the mucus layer, with the addition of PEG to the surface of particulate systems being the current “gold standard” [38]. This strategy has improved the diffusion of particles through the mucus [39] since PEG decreases mucus–nanoparticle interactions [40]. This interesting mucus-penetrating property is related to the non-ionic and hydrophilic character of PEG [39]. Taking this into account, the interaction of mucin with EFV-loaded Soluplus^®^ NMs was evaluated by DLS. Particularly, this technique can be used to estimate the interaction between nanoparticles and mucin based on the increase, or not, of the nanocarrier size [22]. In our case, we were focused on how the PEG surface density of the micelles affected their in vitro stability. Moreover, we employed different SGIFs, taking into account the pH variations along the gastrointestinal tract (Table S1) and its influence on dynamic colloidal dispersions such as polymeric micelles. Hence, micellar dispersions were incubated with a mucin dispersion and particle size was determined at 37 °C. As shown in Figure S1, the micellar size distribution of the mucin/EFV-loaded Soluplus^®^ NM mixture remains in the same size range as the EFV nanomicelles alone with unimodal size distribution, regardless of the final pH value. For instance, the EFV-NMs showed size values between 117.7 ± 7.7 nm (pH 1.2) and 147.8 ± 5.7 nm (pH 6.0). These values remained unchanged after the addition of mucin (0.025% *w*/*v*). In this case, the size values were 118.3 ± 10.1 nm (pH 1.2) and 151.8 ± 3.4 nm (pH 6.0). On the contrary, the control of mucin alone (0.025% *w*/*v*) exhibits a different size range, between 292.7 ± 41.1 and 465.8 ± 29.8 nm for the SGIFs assayed (Figure 3). All these results clearly suggest that the PEG-based micellar corona of the Soluplus^®^ NMs represents a key strategy to overcome the adhesive properties of mucin. No micellar size variations were observed after their combination with mucin. This behavior is probably due to the presence of PEG 6000 in the structure of Soluplus^®^ copolymer, which has been shown to have intense mucus-permeating properties [41]. This represents a potential key clinical feature for our EFV nanomicelles to improve the oral pharmacotherapy of ZIKV, DENV and YFV infections, overcoming the GI mucus barrier.

### 3.4. Micellar Stability Studies

In order to gain further insight into the reproducibility of the formulation process, the size distribution variability between five different batches of EFV-NMs was evaluated. As can be observed in Table S2, the NMs exhibited minimal batch-to-batch size variation, with a unimodal size distribution between batches (Figure S2). Slight size variations can be attributed to the dynamic nature of the NMs [32]. A similar trend was observed for the PDI values, denoting an acceptable reproducibility of the formulation process. For instance, PDI values ranged between 0.10 and 0.12 for every batch assayed (Table S2).

One of the most important critical parameters in the pharmaceutical development of polymeric NMs is colloidal stability over time [39,42], given that the dynamic nature of these systems can favor the process of disassembly and the premature release of the encapsulated drug [32].

Considering this, the physicochemical stability of liquid EFV-NMs at 4 °C (refrigerator temperature) was studied over 24 months. The results indicated that the NMs maintained an adequate and constant size at refrigerator temperature during the 2 years of storage (Table 3). Also, the EFV content of the nanoformulation remained practically unchanged over time, as the drug content was 96.3 ± 2.6% after 2 years of storage. Interestingly, the NMs demonstrated an excellent colloidal stability in aqueous media. The Z-ave values for the drug-free micelles were 87.0 ± 0.3 nm and 85.9 ± 2.1 nm at 0 and 24 months, respectively, with narrow PDI values (Table 3). Following a similar trend, EFV encapsulation within the Soluplus^®^ NMs did not modify their colloidal stability. Indeed, the EFV-NM size values remained almost unchanged in distilled water over time. For instance, the nanoformulation exhibited size values of 104.5 ± 10.6 nm and 107.7 ± 0.2 nm after 1 and 24 months, respectively. These results suggest the absence of micellar aggregation over time under the storage condition (distilled water, 4 °C), which could be associated with the low CMC value of the micellar-forming biopolymer (Soluplus^®^) [21]. Taking into account the dynamic nature of NMs, these results also demonstrated that EFV did not affect the colloidal stability of Soluplus^®^ NMs over time.

Altogether, these findings highlight the in vitro physicochemical stability of our micellar nanoformulation, where EFV-NMs can be stored as a liquid formulation at 4 °C for over 2 years.

It is worth stressing that this is the first time the in vitro physical stability of the Soluplus^®^ NMs in water over 24 months has been reported, to the best of our knowledge.

### 3.5. In Vitro EFV Release

**Figure 4:**
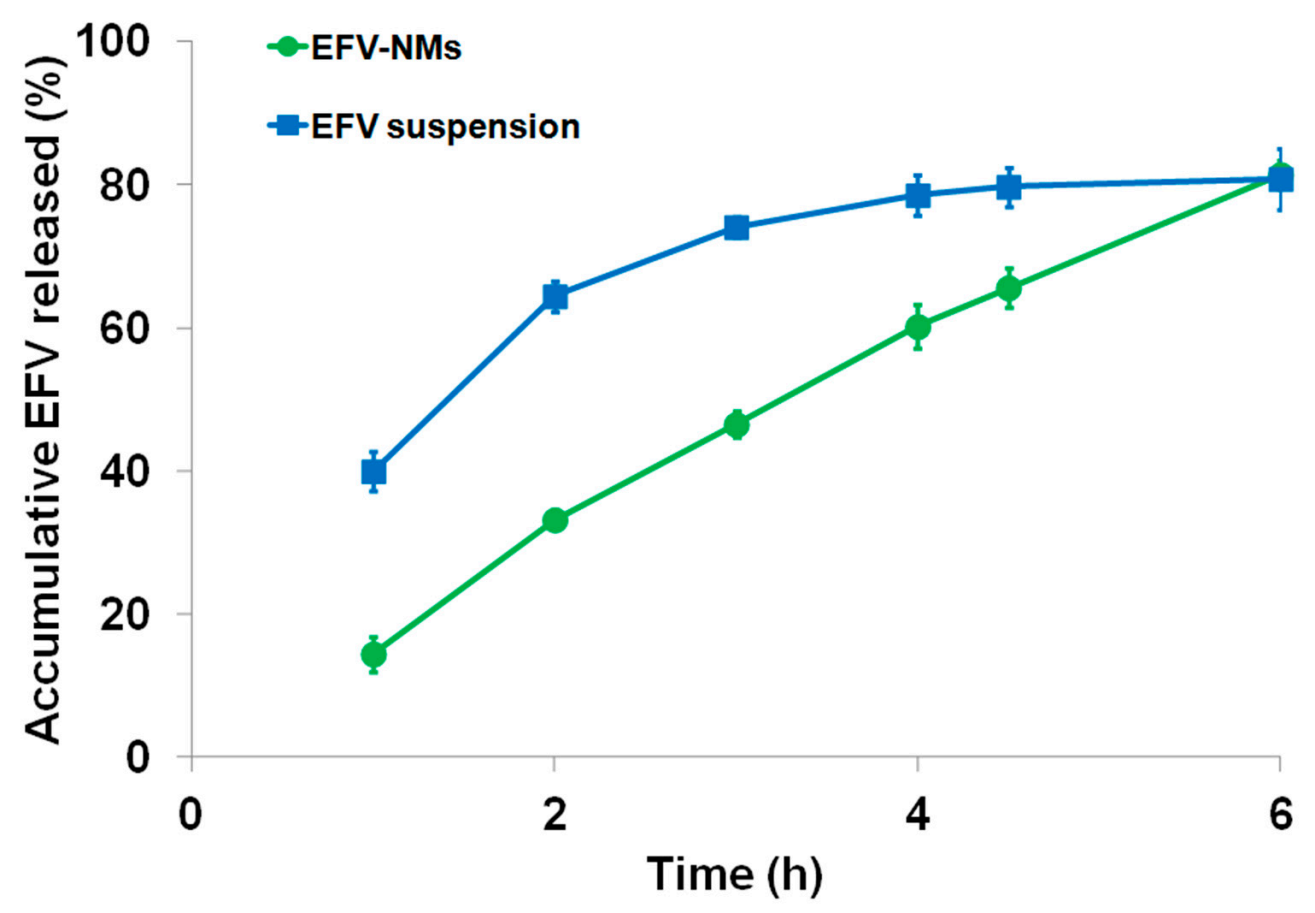
In vitro EFV release profiles from EFV-NMs and EFV suspension at 37 °C over 6 h. Data are means ± standard deviation (S.D.), *n* = 3.

In order to evaluate the EFV’s in vitro release, we compared the drug release profile between the EFV (10 mg/mL)-loaded 7% Soluplus^®^ NMs and EFV (10 mg/mL)suspension prepared with the commercial tablets (VIRORREVER 600) in SGIFs at body temperature (37 °C) over 6 h (Figure 4). To assess Sink conditions, between 80 and 30% *v*/*v* of ethanol was added to the release mediums [19]. For each drug molecule to be collected in the release medium, it had to first be released from the micelle and then diffuse across the dialysis membrane. In the case of the suspension, the drug must first be dissolved inside the bag and then pass through the membrane. As shown in Figure 4, the in vitro release assay indicated that the EFV release from polymeric micelles was much slower and sustained compared to the drug suspension. For example, the EFV-NMs and EFV suspension exhibited drug releases of 33% and 64% at 2 h and 60% and 78% at 4 h, respectively. Finally, ~80% of the EFV was released by both formulations at 6 h. In the evaluated period, the in vitro release of EFV from the NMs follows a zero-order profile (R^2^ = 0.9876). A similar behavior was observed for ibuprofen-loaded NMs and ibuprofen suspension, where the nanomicellar system presented a sustained release profile [29]. This behavior is due to the interaction between the poorly soluble drugs and the hydrophobic chains of Soluplus^®^ allowing a sustained release pattern [19,29].

### 3.6. In Vitro Cytocompatibility

The in vitro cytotoxicity of EFV, the EFV-NMs and drug-free NMs was comparatively evaluated after 48 h of treatment in a human lung cancer cell line (A549) and a monkey kidney epithelial cell line (Vero), which are appropriate systems for flavivirus multiplication [43].

**Figure 5:**
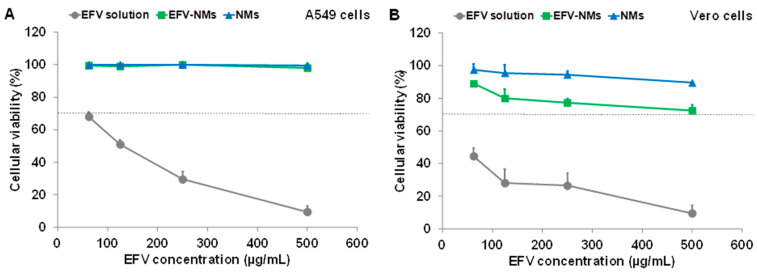
Cell viability of (**A**) A549 cells and (**B**) Vero cells after 48 h of treatment with EFV solution (10 mg/mL), drug-free NMs and EFV-NMs (10 mg/mL) (37 °C, 5% CO_2_). Data are means ± standard deviation (S.D.) (*n* = 3).

On the one hand, cell viability was not affected by the EFV-loaded NMs in the range of concentrations tested in both cell lines. A similar profile was observed by the drug-free NMs as shown in Figure 5.

On the other hand, EFV exhibited a dose-dependent cytotoxicity in both cell types. The improved effect on cell survival of the nanomicellar system after EFV encapsulation compared to the free drug was reflected by the CC_50_ values (Table 4). For example, the EFV-NMs’ CC_50_ values were 8- and 17-fold higher than those of the EFV solution in A549 and Vero, respectively (Table 4). A similar behavior was also recently observed in a Caco-2 cell line [19].

### 3.7. In Vitro Antiviral Efficacy

The pursuit of effective antiviral drugs is vital to mitigate the spread of emerging viral infections [44]. The design of new antiviral molecules is an expensive and long process [45]. Drug repositioning is a strategy that may offer an interesting opportunity to provide an effective pharmacotherapeutic treatment to combat these diseases, ensuring that medicines are accessible to everyone in a cost-effective manner [44]. In the case of EFV, it is important to highlight that it has been available as a low-cost antiretroviral since the expiration of its patent [46].

Taking into account the aforementioned factors, the antiviral activity of EFV and the EFV-NMs was explored in A549 and Vero cells which were infected with DENV-2, ZIKV or YFV (m.o.i. 0.1). After 1 h of adsorption at 37 °C, both infected cell lines were treated with serial non-cytotoxic dilutions of EFV-NMs, EFV or NMs, respectively. After 48 h of infection, supernatants were collected, and the viral yield was titrated by plaque assay.

**Figure 6:**
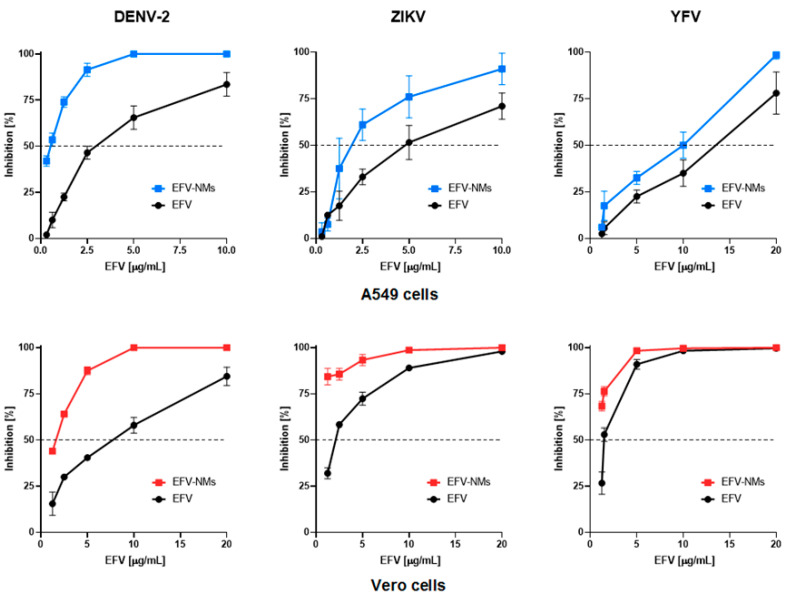
Antiviral activity. A549 and Vero cells were infected with DENV-2, ZIKV or YFV (m.o.i. 0.1) in the absence of compound and after 1 h adsorption at 37 °C the inocula were removed and the cells were re-fed with MM containing, or not containing (VC), different concentrations of EFV and EFV-NMs. The antiviral activity of the compounds was determined at 48 h p.i.; supernatant cultures were harvested and the viral yields were determined by plaque assays. All determinations were performed in triplicate. The percentages of inhibition are presented as the mean ± SD obtained from three independent experiments.

EFV demonstrated significant antiviral activity against DENV-2, ZIKV and YFV, with a dose-dependent increase in the percentage of inhibition in both cell lines at non-cytotoxic concentrations (Figure 6). The EFV solution presents EC_50_ values between 1.48 and 12.60 µg/mL for each viral infection (Table 4). In the case of ZIKV, these results are consistent with previous results, where EFV showed EC_50_ values in Vero cells between 8.14 and 9.60 µg/mL [15]. To the best of our knowledge, this is the first report that shows the in vitro EFV antiviral activity in DENV-2 and YFV infections.

**Figure 7:**
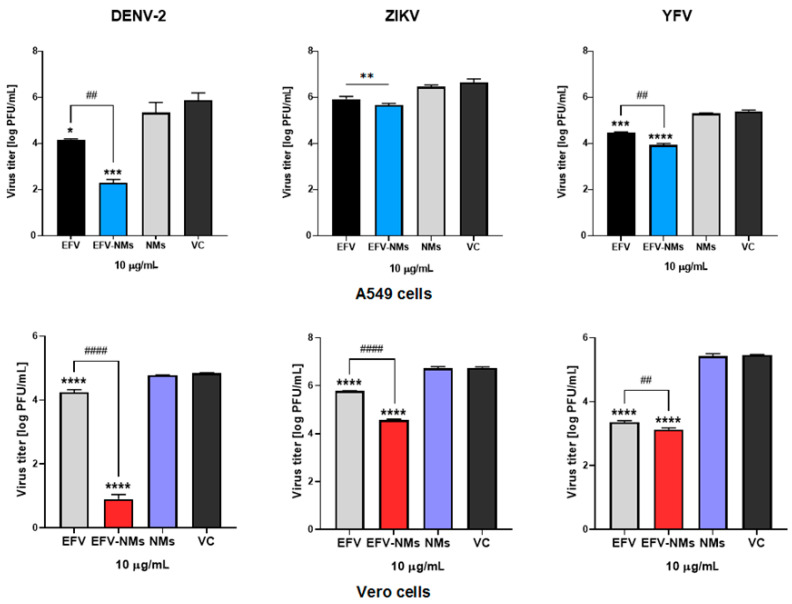
Viral titer inhibition. A549 and Vero cells were infected with DENV-2, ZIKV or YFV (m.o.i. 0.1) in the absence of compound and, after 1 h adsorption at 37 °C, re-fed with MM containing, or not containing (VC), a single concentration (10 µg/mL) of EFV, EFV-NMs or NMs. The antiviral activity of the compounds was determined after 48 h p.i.; supernatant cultures were harvested and the viral yields were determined by plaque assays. All determinations were performed in triplicate and the viral titer values are presented as the mean ± SD obtained from three independent experiments. *p*-values were determined using an ANOVA analysis followed by Dunnett’s post hoc test. Hashtag represents significant differences with respect to the treatments, asterisk represents significant differences with respect to the VC: * *p* < 0.05; ##/** *p* < 0.01; *** *p* < 0.001; ####/**** *p* < 0.0001.

Moreover, the EFV-NMs exhibited significantly more antiviral activity compared with the EFV solution in all tested flaviviruses. This behavior is reflected in the lower EC_50_ values for the EFV-NMs versus the EFV solution in both cell lines and for each viral infection (Table 4), thus demonstrating a greater antiviral potency by the nanomicellar system. Similar results were observed for lamivudine stearate (LAS)-loaded stearic acid-g-chitosan oligosaccharide micelles, where the inhibitory effect of LAS on the hepatitis B virus was enhanced after its encapsulation in the micelles [47]. This effect also was evidenced by the viral titer reduction. This assay was conducted using a fixed concentration of EFV (10 µg/mL) and a 48 h post-infection treatment with an m.o.i. of 0.1. Viral titers were subsequently determined via the PUF method. In this assay, it was observed that for each cell line and each viral infection the reduction in viral titer by the EFV-NMs was significantly greater than that of the EFV solution (Figure 7). The most pronounced difference was observed for the DENV-2 infection (Figure 7), representing a reduction of 1.8 and 4.7 log in A549 and Vero cells, respectively. In the case of ZKV, the reduction was of 1.05 and 1.27 log for A549 and Vero cells, respectively, and for YFV, 1.14 and 1.07 log in A549 and Vero cells, respectively. Notably, it is worth mentioning that for the treatments with drug-free NMs, no antiviral effect was observed.

It is well known that EFV is a non-nucleoside inhibitor that targets the reverse transcriptase enzyme of the human immunodeficiency virus (HIV) [14]. Further, it has been studied that non-nucleoside inhibitors are able to inhibit the enzymatic function of a promising antiviral drug target known as NS5 protein [48,49]. Indeed, EFV has demonstrated affinity to ZIKV target proteins along with the in vitro inhibition of ZIKV replication [15]. Despite this, future studies will be necessary to elucidate its anti-flavivirus mechanism.

An early in vitro indicator that can predict the probability of a compound having therapeutic value is the selectivity index (SI) [50]. This parameter is defined as the relationship between the toxic concentration of a drug and its effective antiviral concentration (CC_50_/EC_50_) [51]. Taking this into account, a compound that presents cytotoxicity at high concentrations and that has antiviral activity at low concentrations is desired. As we have observed for the antiviral activity, the SI values reveal that the EFV-NMs exhibited considerably more biological efficacy compared to the EFV solution in both cell lines and for each viral infection (Table 4). It is generally considered that compounds with SI values ≤ 1 are considered toxic, ≥5 as acceptable, and ≥10 as a selective bioactive compound [52,53,54]. In our case, the EFV solution presented SI values > 10 in most of the cases, except for DENV-2 in Vero cells (SI = 7), while the EFV-NMs showed SI values greater than 100 in all cases. These results demonstrate the very good in vitro performance of EFV as an antiviral drug and the excellent performance of the nanoformulation against these flaviviruses.

Last but not least, the EC_50_ values achieved by EFV and the EFV-NMs were comparable, and higher than the C_max_ value reported for EFV in humans (4 μg/mL) [55]. Interestingly, the EFV-NMs showed EC_50_ values < EFV C_max_ in most of the cases, except for YFV in A549 cells (Table 4). On the other hand, the EFV solution presented EC_50_ values < EFV C_max_ for ZIKV and YFV in Vero cells and for DENV-2 in A549 cells (Table 4). In the other cases, the EC_50_ values were between 5 and 12.6 µg/mL. However, it is important to note that EFV plasma concentrations can vary between 9.5 and 15.8 µg/mL in ~40% of patients, and can reach 25.3 µg/mL in some cases [56]. Taking this into account, the antiviral activity of EFV could be compatible with the recommended oral dose for adults (600 mg) employed in the treatment of HIV.

Despite the difficulties in translating in vitro results into clinical application, these results highlight the potential of our EFV nanoformulation for clinical use in the treatment of these viral infections.

In the case of dengue, different anti-DENV drugs candidates have not shown significant benefits in clinical trials [57]. In this way, our EFV micellar formulation represents an excellent nanotechnological approach for EFV repurposing against DENV.

### 3.8. In Vivo Pharmacokinetic Study

It is well established that EFV has poor biopharmaceutical properties which could negatively affect its oral bioavailability. This is the main reason why we studied the oral performance of our EFV nanoformulation in Wistar rats. Further, the results were compared with those obtained for an extemporaneous EFV suspension. Pharmacokinetic parameters such as maximum plasma concentration (C_max_), time to maximum plasma concentration (t_max_), area under the curve between 0 and 8 h (AUC_0–8_) and plasma elimination half-life (t½) were investigated using an EFV dose of 40 mg/kg. These EFV parameters were obtained by the non-compartmental analysis of plasma concentrations for every time point. As cam be observed in Table 5, C_max_ values significantly (*p* < 0.05) increased (1.64-fold) from 4800 ng/mL (EFV suspension) to 7879 ng/mL for the Soluplus^®^ NMs. Following a similar trend, the AUC_0–8_ values for the NMs (43,794 ng/mL/h) were significantly (*p* < 0.01) higher than those observed for an EFV suspension (21,233 ng/mL/h). All together, these results represent a 2.06-fold increment on oral EFV bioavailability (F_r_: 206.3%) (Table 5). A similar behavior was observed for poloxamer and poloxamine micelles, where the EFV encapsulation produced an improvement in the drug’s pharmacokinetic parameters compared to an EFV suspension at a 40 mg/kg dose [16,18].

On the other hand, pharmacokinetic parameters such as t_max_ and the elimination half-life of EFV did not show significant differences between the drug suspension and the EFV-NMs (Table 5). The latter could suggest that EFV encapsulation within NMs did not affect the time needed to reach the plasmatic maximum concentration of the drug and its elimination.

Therefore, the oral administration of this nanoformulation, which is composed of a PEG6000-based micellar corona, could enhance EFV’s pharmacokinetic parameters, this being consistent with the results obtained.

### 3.9. Histological Evaluation of Stomach, Small Intestine and Large Intestine Segments

**Figure 8:**
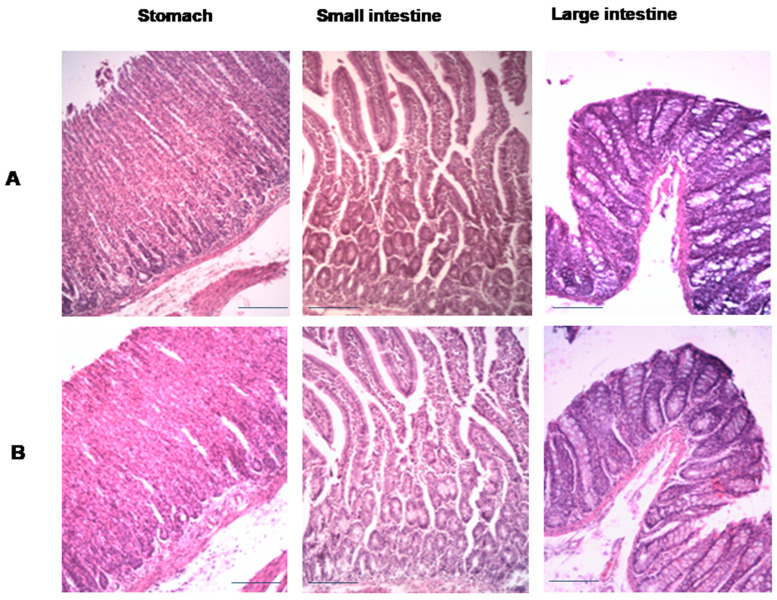
Representative images of hematoxylin and eosin (H&E) staining of stomach, small and large intestine treated with (**A**) saline solution and (**B**) EFV-NMs (magnification: ×100; scale bar = 100 µm). Representative sections from three rats analyzed for each condition.

Research has shown that antiviral treatments can be effective in controlling viral infection and replication. The duration of these treatments may vary depending on the type of viral infection. In the case of DENV, ZIKV and YFV infections, antiviral treatment could be at least one-week long [58,59,60]. Taking this into account, EFV-NMs were administered once a day for a week. Once the treatment finished, histological studies were performed to assess pathological changes in the epithelium of the stomach, small intestine and large intestine. The EFV-NM group showed no morphological changes in the mucosa of the stomach, small intestine and large intestine (Figure 8) compared to the control group. Furthermore, no signs of necrosis were observed in the tissues and, particularly, no evidence of damage to villi in the small intestine was found. A similar behavior was observed for a Soluplus^®^-based 9-nitrocamptothecin solid dispersion, where this oral dosage form demonstrated biocompatibility by not causing damage in the gastrointestinal segments [61].

It is important to mention that Soluplus^®^ presents an oral lethal dose 50% (LD50) of >5 g/kg and a No-Observed-Effect Level (NOEL) of 2 g/kg/day in rats [62]. In our case, the daily dose of EFV (40 mg/kg) administered corresponds to a Soluplus^®^ daily dose of 280 mg/kg; therefore, the oral administration of this formulation was expected not to produce toxicity, as is consistent with the results obtained.

These results showed a very good safety profile for the EFV-loaded Soluplus^®^ NMs in the gastrointestinal tract.

## 4. Conclusions

In the present study, we successfully developed an oral EFV nanomicellar formulation with an excellent in vitro cytocompatilibility in A549 and Vero cells. It is important to highlight that treatment with EFV, employed as a drug repositioning strategy, significantly inhibited flaviviruses DENV-2, ZIKV and YFV replication in cell culture in a dose-dependent manner, and it may be proposed as a potentially effective approach for combating these viral infections. Furthermore, its encapsulated form in Soluplus^®^ polymeric micelles enhances its in vitro antiviral activity, significantly reduces cytotoxicity in A549 and Vero cell lines, and, thus, presents a more selective treatment against these viruses. In vivo studies also demonstrated that EFV encapsulation within Soluplus^®^ micelles significantly enhanced the drug’s relative oral bioavailability compared with a suspension dosage form, with an absence of necrosis and morphological changes along the gastrointestinal mucosa.

Overall, our EFV nanoformulation exhibits great potential as a nanotechnological platform for optimized ZIKV, DENV and YFV antiviral therapy. Future studies will be focused on the antiviral efficacy of EFV and EFV-NMs in animal models.

## Figures and Tables

**Table 1:** Main parameters used to quantify efficiency of EFV solubilization in distilled water at 25 °C.

Soluplus^®^ Concentration (% *w*/*v*)	S_a_ (mg/mL) (±S.D.)	F_s_	S_a_ (mM) (±S.D.)	X	P	PM	∆Gs (KJ/mol)
1	1.86 (0.55)	464	5.76 (1.74)	69.4	454	5368	−21.29
3	5.31 (0.75)	1328	16.82 (2.38)	66.2	1327	5220	−21.22
5	8.99 (1.12)	2246	28.48 (3.55)	67.2	2247	5303	−21.26
7	14.67 (1.56)	3668	46.47 (4.94)	78.4	3667	6181	−21.64
10	19.21 (2.11)	4803	60.85 (6.68)	71.8	4802	5666	−21.42

S_a_: apparent solubility; F_s_: solubility factor; X: molar solubilization capacity; P: partition coefficient; PM: molar partition coefficient; ∆Gs: free energy of solubilization.

**Table 2:** EFV payload, micellar size and size distribution of drug-free and EFV-loaded (10 mg/mL) Soluplus^®^ nanomicelles (7% *w*/*v*) at 25 and 37 °C. Results are expressed as mean ± standard deviation (S.D.) (*n* = 3).

Samples	Size				DL (%)	EE (%)
	25 °C		37 °C			
	Z-Ave (nm) (±S.D.)	PDI (±S.D.)	Z-Ave (nm) (±S.D.)	PDI (±S.D.)		
Drug-free NMs	83.4 (0.2)	0.12 (0.01)	2363.0 (247.2)	1.00 (0.01)	-	-
EFV-NMs	95.8 (0.3)	0.19 (0.01)	85.4 (0.4)	0.20 (0.01)	12.4 (±0.1)	99.3 (±0.8)

**Table 3:** Storage stability of EFV (10 mg/mL)-loaded Soluplus^®^ (7% *w*/*v*) NMs at 4 °C over 2 years.

Time (Months)	Size			
	Drug-Free NMs		EFV-NMs	
	Z-Ave (nm) (±S.D.)	PDI (±S.D.)	Z-Ave (nm) (±S.D.)	PDI (±S.D.)
0	87.0 (0.3)	0.17 (0.01)	95.8 (0.3)	0.19 (0.01)
1	84.0 (2.1)	0.13 (0.01)	104.5 (10.6)	0.19 (0.02)
6	83.2 (0.5)	0.12 (0.01)	104.2 (5.0)	0.21 (0.02)
12	84.9 (0.1)	0.14 (0.01)	103.8 (8.6)	0.20 (0.01)
24	85.9 (2.1)	0.16 (0.02)	107.7 (0.2)	0.23 (0.01)

**Table 4:** Cytotoxicity and antiviral activity of EFV solution and EFV-NMs on A549 and Vero cell lines. CC_50_ and EC_50_ values are represented as means ± SD, *n* = 3.

System	Cell Line	CC_50_ (µg/mL) (±S.D.)	Virus					
			DENV-2		ZIKV		YFV	
			EC_50_ (µg/mL) (±S.D.)	SI	EC_50_ (µg/mL) (±S.D.)	SI	EC_50_ (µg/mL) (±S.D.)	SI
EFV solution	A549	126.0 (2.9)	2.69 (1.16)	46.8	4.98 (0.04)	25.3	12.60 (0.43)	10.0
	Vero	56.8 (11.0)	8.17 (0.85)	7.0	2.41 (0.05)	23.6	1.48 (0.03)	38.4
EFV-NMs	A549	>1000	0.58 (0.03)	>1724	1.48 (0.02)	>676	10.00 (1.15)	>100
	Vero	>1000	2.46 (0.01)	>407	<1.25	>800	<1.25	>800

CC_50_ = cytotoxic concentration 50 (CC_50_); EC_50_ = effective concentration 50 (EC_50_); SI = selectivity index.

**Table 5:** The pharmacokinetic parameters of the EFV suspension and the EFV-NMs administered orally (EFV dose: 40 mg/kg). The EFV formulation concentration was 10 mg/mL. The results are expressed as means ± S.E. (*n* = 5).

Parameter	Units	EFV Suspension	EFV-NMs
t½	h	5.97 ± 1.68	6.98 ± 1.58
t_max_	h	3.2 ± 0.2	3.2 ± 0.2
C_max_	ng/mL	4800 ± 703	7879 ± 1525 ^#^
AUC_0–t_	ng/mL/h	21,233 ± 2378	43,794 ± 7807 ^&^
AUC_0–∞_	ng/mL/h	41,454 ± 9387	88,415 ± 10,895 ^&^
F_r_	%	100.0	206.3

^#^ *p* < 0.05 vs. EFV suspension; ^&^ *p* < 0.01 vs. EFV suspension.

## Data Availability

Data will be made available on request.

## Supplementary Materials

The following supporting information can be downloaded at: https://www.mdpi.com/article/10.3390/pharmaceutics17020241/s1, Figure S1: Particle size distribution for EFV-NMs in absence and presence of 0.025% *w*/*v* mucin in different simulated gastrointestinal fluids (pH 1.2, 6.8, 7.5 and 6.0); Figure S2. Size distribution of five EFV-NMs batches at 25 °C; Table S1: Details of four mediums employed simulating gastrointestinal segments for in vitro assays; Table S2: Size and size distribution of five EFV-NM batches at 25 °C.

## Abbreviations

The following abbreviations are used in this manuscript:

WHO	World Health Organization
DENV	Dengue virus
ZIKV	Zika virus
YFV	Yellow fever virus
EFV	Efavirenz
NMs	Nanomicelles
SGIFs	Simulated gastrointestinal fluids
EFV-NMs	Efavirenz-loaded nanomicelles
DLS	Dynamic light scattering
TEM	Transmission electron microscopy
SGF	Simulated gastric fluids
SIF	Simulated intestinal fluids
SCF	Simulated colonic fluids
SI	Selectivity index
CC_50_	Cytotoxic concentration 50
EC_50_	Effective concentration 50
AUC	Area under the curve
C_max_	Maximum plasma concentration
t_max_	Time to maximum plasma concentration
F_r_	Relative oral bioavailability
