# Supplementary material for: Efavirenz Repurposing Challenges: A Novel Nanomicelle-Based Antiviral Therapy Against Mosquito-Borne Flaviviruses
Source: Pharmaceutics. 2025 Feb 12;17(2):241. doi: 10.3390/pharmaceutics17020241 (PMC11859092; doi:10.3390/pharmaceutics17020241)
Supplement: Supplementary file 1 [file pharmaceutics-17-00241-s001.zip › pharmaceutics-3441547-supplementary.pdf]

Efavirenz Repurposing Challenges: A Novel Nanomicelle-Based Antiviral Therapy Against Mosquito-Borne Flaviviruses

Sofía Maldonado [1,2,3], Pedro Fuentes [1,2], Ezequiel Bernabeu [1,2,3], Facundo Bertera [2,4], Javier Opezzo [2,4], Eduardo Lagomarsino [2,5], Hyun J. Lee [6], Fleming Martínez Rodríguez [7], Marcelo R. Choi [6,8], María Jimena Salgueiro [2,9], Elsa B. Damonte [10], Christian Höcht [2,4], Marcela A. Moretton [1,2,3,*], Claudia S. Sepúlveda [10] and Diego A. Chiappetta [1,2,3]

[1]Universidad de Buenos Aires, Facultad de Farmacia y Bioquímica, Cátedra de Tecnología Farmacéutica I, Buenos Aires, Argentina.

[2]Universidad de Buenos Aires, Instituto de Tecnología Farmacéutica y Biofarmacia (InTecFyB), Buenos Aires, Argentina.

[3]Consejo Nacional de Investigaciones Científicas y Técnicas (CONICET).

[4]Universidad de Buenos Aires, Facultad de Farmacia y Bioquímica, Cátedra de Farmacología, Buenos Aires, Argentina.

[5]Universidad de Buenos Aires, Facultad de Farmacia y Bioquímica, Cátedra de Farmacia Clínica, Argentina.

[6]Universidad de Buenos Aires. Facultad de Farmacia y Bioquímica. Departamento de Ciencias Biológicas, Cátedra de Anatomía e Histología. Buenos Aires, Argentina

[7]Facultad de Ciencias, Departamento de Farmacia, Grupo de Investigaciones Farmacéutico-Fisicoquímicas, Universidad Nacional de Colombia, Sede Bogotá, Colombia.

[8]Universidad de Buenos Aires. CONICET. Instituto Alberto C. Taquini de Investigaciones en Medicina Traslacional (IATIMET), Buenos Aires, Argentina

[9]Universidad de Buenos Aires, Facultad de Farmacia y Bioquímica, Cátedra de Física, Buenos Aires, Argentina.

[10]Instituto de Química Biológica de la Facultad de Ciencias Exactas y Naturales (IQUIBICEN), CONICET-Universidad de Buenos Aires, Buenos Aires, Argentina.

*Corresponding author

Prof. Dr. Marcela A. Moretton

Departamento de Tecnología Farmacéutica, Facultad de Farmacia y Bioquímica, Universidad de Buenos Aires, 956 Junín St., 6th Floor, Buenos Aires CP1113, Argentina

Email: mmoretton@ffyb.uba.ar

Phone: +54-11-5287-4633

In vitro micellar interaction with mucin

The interaction of mucin with EFV-loaded Soluplus® NMs was evaluated by DLS. In our case, we were focused on how the PEG surface density of the micelles affects their *in vitro* stability. Moreover, we employed different SGIFs taking into account the pH variations along the gastrointestinal tract (**Table S1**) and its influence on dynamic colloidal dispersions as polymeric micelles. Hence, micellar dispersions were incubated with mucin dispersion and particle size was determined at 37 ºC. As shown in **Figure 3**, micellar size distribution of the mixture mucin/ EFV-loaded Soluplus® NMs remains in the same size range of the EFV-nanomicelles alone with unimodal size distribution, regardless the final pH value. For instance, EFV-NMs showed size values between 117.7 ± 7.7 nm (pH 1.2) and 147.8 ± 5.7 nm (pH 6.0). These values remained unchanged after the addition of mucin (0.025 %w/v). In this case size values were 118.3 ± 10.1 nm (pH 1.2) and 151.8 ± 3.4 nm (pH 6.0). On the contrary, the control of mucin alone (0.025 %w/v) exhibit a different size range between 292.7 ± 41.1 and 465.8 ± 29.8 nm for the SGIFs assayed (**Figure 3**). All these results clearly suggest that the presence of PEG in the surface of the polymeric micelles represents a key strategy to overcome the adhesive properties of the mucin. Then, no micellar size variations were observed after their combination with mucin (**Figure S1**).

Table S1. Details of the four mediums employed simulating gastrointestinal segments for *in vitro* assays.

Medium	Mimic gastrointestinal segment	pH
1	Stomach (SGF)	1.2

2	Jejunum (SIF pH 6.8)	6.8
3	Ileum (distal) (SIF pH 7.5)	7.5
4	Colon (proximal) (SCF)	6.0

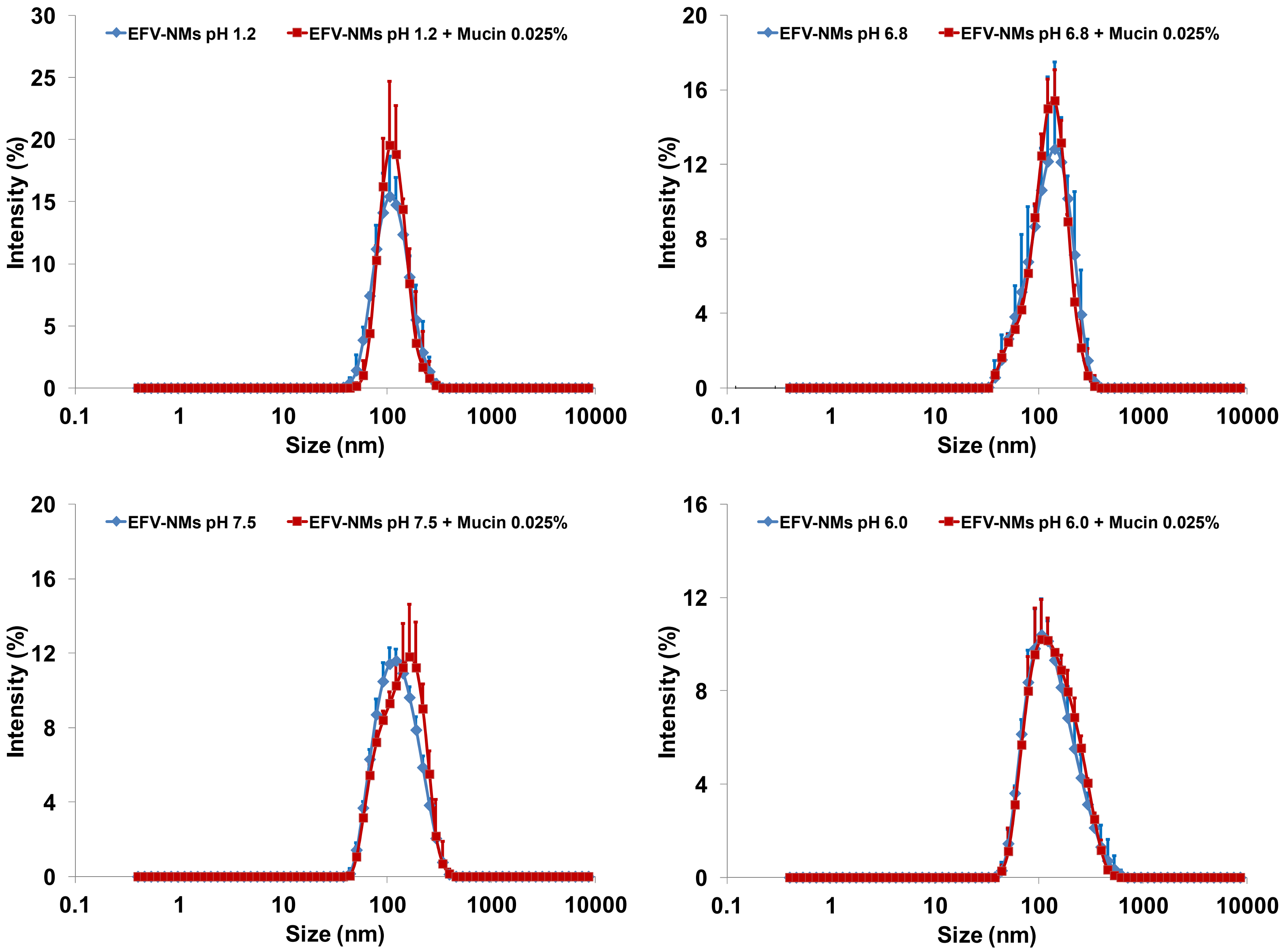

Figure S1. Particle size distribution for EFV-NMs in the absence and presence of 0.025 % w/v mucin in different simulated gastrointestinal fluids (pH 1.2, 6.8, 7.5 and 6.0)

Micellar stability studies

In order to gain further insight in the reproducibility of the formulation process, the size distribution variability between 5 different batches of EFV-NMs was evaluated. As it could be observed in **Table S2**, NMs exhibited minimal batch-to-batch size variation with an unimodal size distribution between batches (**Figure S2**). Slight size variations could be attributed to the dynamic nature of NMs [**Owen 2012**]. A similar trend was observed for the PDI values denoting an acceptable reproducibility of the formulation process. For instance, PDI were ranged between 0.10 and 0.12 for every batch assayed (**Table S2**).

Table S2. Size and size distribution of five EFV-NMs batches at 25 ºC.

**Formulation**	**Batch**	**Size**	
		**Z-ave (nm) (±S.D.)**	**PDI (±S.D.)**
EFV-NMs	1	102.7 (0.5)	0.11 (0.02)
	2	98.0 (0.1)	0.11 (0.01)
	3	109.0 (0.6)	0.12 (0.02)
	4	99.0 (0.8)	0.11 (0.02)
	5	95.2 (0.7)	0.10 (0.01)

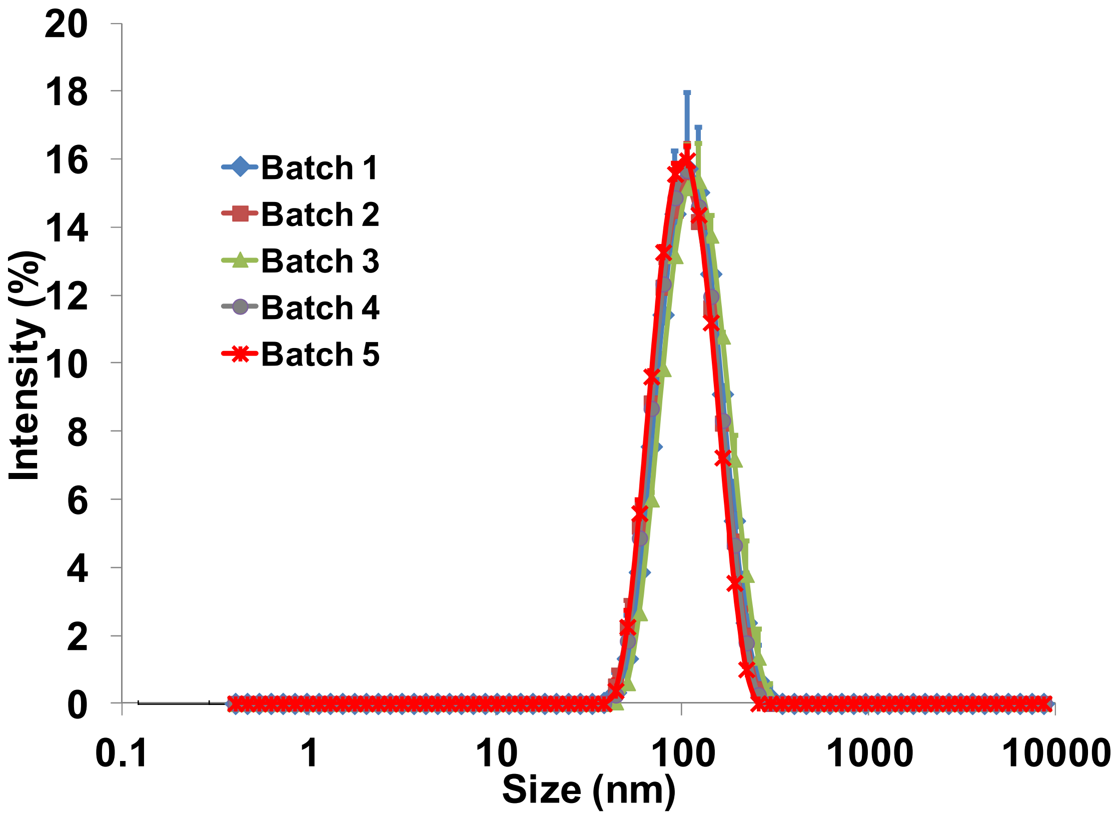

Figure S2. Size distribution of five EFV-NMs batches at 25 °C.
